# A new crystal form of Lys48-linked diubiquitin

**DOI:** 10.1107/S1744309110027600

**Published:** 2010-08-21

**Authors:** Jean-François Trempe, Nicholas R. Brown, Martin E. M. Noble, Jane A. Endicott

**Affiliations:** a Department of Biochemistry, McGill University, 3649 Promenade Sir William Osler, Montreal, Québec H3G 0B1, Canada; b Laboratory of Molecular Biophysics, Department of Biochemistry, South Parks Road, Oxford OX1 3QU, England

**Keywords:** ubiquitin, diubiquitin, Lys48, isopeptide bonds, UBA

## Abstract

Lys48-linked polyubiquitin chains are recognized by the proteasome as a tag for the degradation of the attached substrates. Here, a new crystal form of Lys48-linked diubiquitin (Ub_2_) was obtained and the crystal structure was refined to 1.6 Å resolution. The structure reveals an ordered isopeptide bond in a *trans* configuration. All three molecules in the asymmetric unit were in the same closed conformation, in which the hydrophobic patches of both the distal and the proximal moieties interact with each other. Despite the different crystallization conditions and different crystal packing, the new crystal structure of Ub_2_ is similar to the previously published structure of diubiquitin, but differences are observed in the conformation of the flexible isopeptide linkage.

## Abstract 2

A new crystal form of Lys48-linked diubiquitin was obtained and its structure was determined by X-ray crystallography to 1.6 Å resolution.

## 1. Introduction

The ubiquitin–proteasome pathway is a fundamental cellular process in eukaryotes that controls protein degradation. Substrates are tagged with ubiquitin through a cascade of enzymatic reactions that is initiated by the activation of ubiquitin by the E1 enzyme, followed by ubiquitin conjugation to E2 and finally transfer of the activated ubiquitin from E2 to a specific substrate *via* an E3 ligase (Hershko & Ciechanover, 1998 ▶). Ubiquitin molecules are assembled through the formation of an isopeptide bond between the carboxyl-terminal group of ubiquitin and the side-chain ∊-amino group of a lysine in another ubiquitin molecule (termed the distal and proximal moieties, respectively) or on the substrate. The 26S proteasome is able to recognize and degrade substrates tagged with a Lys48-linked polyubiquitin chain (Finley, 2009 ▶).

Several proteasomal ubiquitin receptors have been described, including the 19S regulatory particle base subunits S5a/Rpn10 (Deveraux *et al.*, 1994 ▶) and Rpn13 (Husnjak *et al.*, 2008 ▶), as well as the UBL-UBA-containing proteins HHR23/Rad23, Dsk2/Dph1 and Ddi1/Mud1 (Bertolaet *et al.*, 2001 ▶; Wilkinson *et al.*, 2001 ▶). The interactions of ubiquitin receptors with Lys48-linked polyubiquitin have been characterized at the structural level (Schreiner *et al.*, 2008 ▶; Trempe *et al.*, 2005 ▶; Varadan *et al.*, 2005 ▶; Zhang, Chen *et al.*, 2009 ▶; Zhang, Wang *et al.*, 2009 ▶), but as yet a crystal structure of a Lys48-linked polyubiquitin chain bound to its receptor has not been reported. In an attempt to obtain the structure of Lys48-linked di­ubiquitin (Ub_2_) bound to the Mud1 UBA domain (Trempe *et al.*, 2005 ▶), cocrystallization trials were performed. Diffracting crystals were obtained, but subsequent structure determination revealed that the crystals were solely composed of Ub_2_. The Ub_2_ subunits in the new crystal structure adopt the closed conformation, as observed in the previous crystal structure (Cook *et al.*, 1992 ▶) and in solution (Varadan *et al.*, 2002 ▶). The packing in the new crystal form differs from that in the previous crystal structure and the structure reveals differences in the conformation of the isopeptide linkage and the loop connecting β1 and β2.

## 2. Materials and methods

### 2.1. Purification and crystallization

Ub_2_ was synthesized *in vitro* as described previously (Piotrowski *et al.*, 1997 ▶; Trempe *et al.*, 2005 ▶). Briefly, the reaction mixture contained 50 m*M* Tris–HCl pH 8.0, 2 m*M* ATP, 5 m*M* MgSO_4_, 0.5 m*M* bovine ubiquitin, 0.5 µ*M* recombinant human His_6_-E1 and 50 µ*M* recombinant budding yeast His_10_-Cdc34. The synthesis reaction was per­formed at 310 K overnight. Bovine ubiquitin was purchased as a lyophilized powder (Sigma–Aldrich), His_6_-E1 ubiquitin-conjugating enzyme was expressed from a recombinant baculovirus in Sf9 insect cells and recombinant His_10_-Cdc34 was expressed in BL21 (DE3) *Escherichia coli* cells from a pET16 expression plasmid. Both His-tagged proteins were purified using Ni–NTA agarose resin (Qiagen). The amino-acid sequence of bovine ubiquitin is identical to that of human ubiquitin and yeast Cdc34 has previously been shown to synthesize Lys48-linked polyubiquitin chains *in vitro* with human E1 (Wu *et al.*, 2002 ▶).

The Ub_2_ purification method was a modification of a previously published protocol (Chen & Pickart, 1990 ▶). After completion, the synthesis reaction mixture was dialysed against 50 m*M* ammonium acetate pH 4.5. The mixture was filtered and loaded at 1.0 ml min^−1^ onto a Mono-S cation-exchange chromatography column (HR 5/5, GE Healthcare). The polyubiquitin chains were then eluted with a linear gradient of 0–0.4 *M* KCl over 60 ml. Elution fractions were collected and further purified by size-exclusion chromatography on a Superdex 75 16/60 column (GE Healthcare) equilibrated in crystallization buffer (20 m*M* Tris–HCl pH 8.0, 50 m*M* NaCl, 0.01% NaN_3_). The purity of the different polyubiquitin chains (Ub_1_, Ub_2_, Ub_3_ and Ub_4_) was assessed by SDS–PAGE. The Ub_2_ concentration was determined using UV absorbance at 276 nm. The Mud1 UBA domain (residues 293–332) was expressed and purified as described previously (Trempe *et al.*, 2005 ▶) and dialyzed against crystallization buffer.

Cocrystallization trials of Mud1 UBA with Ub_2_ were performed at a final concentration of 0.5 m*M* Ub_2_ and 0.5–0.75 m*M* Mud1 UBA using Structure Screens 1 and 2 (Molecular Dimensions). Crystals were grown at 295 K by vapour diffusion using the sitting-drop method (1.0 µl drops). Thin rectangular plate-shaped crystals (∼300 × 100 × 30 µm) were grown in 30% PEG 4000, 0.2 *M* Li_2_SO_4_, 0.1 *M* Tris–HCl pH 8.5 from a 1.5:1 molar ratio of UBA:Ub_2_. Conditions with less or no Mud1 UBA yielded smaller crystals of poor diffraction quality.

### 2.2. Data collection and processing

A crystal was cryoprotected using mother liquor supplemented with 15% ethylene glycol and frozen in liquid nitrogen. Data were collected at 100 K on beamline ID-29 at ESRF, Grenoble. Data-collection statistics are shown in Table 1 ▶. Reflections were indexed and integrated using the program *MOSFLM* (Leslie, 2006 ▶) and the intensities were scaled and merged using *SCALA* (Evans, 2006 ▶).

### 2.3. Structure solution and refinement

The phase problem was solved by molecular replacement using the program *Phaser* (McCoy *et al.*, 2007 ▶). The crystal structure of monoubiquitin (PDB code 1ubq; Vijay-Kumar *et al.*, 1987 ▶) was used as a search model, excluding the flexible residues 73–76. Six copies of ubiquitin were found, giving a solvent content of ∼41%. After rigid-body refinement in *REFMAC*5 (Murshudov *et al.*, 1997 ▶), no additional density was observed that could accommodate the UBA domain. Water molecules were added automatically using *ARP*/*wARP* (Perrakis *et al.*, 1997 ▶). The model was then adjusted in the electron-density map using the program *Coot* (Emsley & Cowtan, 2004 ▶). The bulk solvent was modelled using the Babinet method with a mask. After a few cycles of restrained refinement in *REFMAC*5 and model building, a final model was obtained with good overall geometry and a satisfactory fit to the experimental amplitudes (Table 1 ▶). The distal moieties of the three Ub_2_ molecules in the asymmetric unit were named *A*, *C* and *E* and their respective covalently bound proximal moieties were named *B*, *D* and *F*. The co­ordinates and structure factors were deposited in the Protein Data Bank under accession code 3m3j.

## 3. Results and discussion

**Figure 1:**
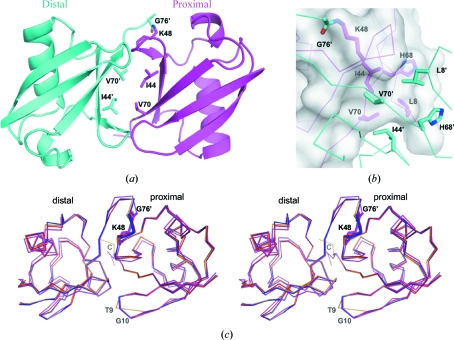
Crystal structure of Lys48-linked Ub_2_. (*a*) Cartoon representation of a Ub_2_ molecule in the crystal structure. The proximal and distal moieties are coloured magenta and cyan, respectively. The atoms forming the isopeptide bond as well as the interface residues Ile44 and Val70 are shown as sticks. Residues labelled with primes belong to the distal moiety. (*b*) Close-up view of the residues forming the interface between the distal and proximal subunits. The molecular surface of the proximal subunit is displayed in transparent white. (*c*) Cross-eye stereoview ribbon display of the overlaid Ub_2_ crystal structures. The three chains in the new crystal structure are shaded yellow, blue and red for *A*–*B*, *C*–*D* and *E*–*F*, respectively. The previously reported crystal structure of Ub_2_ is shaded in magenta (PDB code 1aar; Cook *et al.*, 1992 ▶). Residues that have different conformations in different subunits are labelled. The disordered C-termini of the proximal moieties are labelled ‘C’.

The asymmetric unit of the new crystal form contained three Ub_2_ molecules, which all adopt the same conformation in which the hydrophobic patches of the proximal and distal ubiquitin moieties, centred around Ile44, interact with each other (Fig. 1 ▶ *a*). Most ubiquitin-binding domains interact with the hydrophobic patch of ubiquitin (Hicke *et al.*, 2005 ▶) and thus the conformation in which the patch is buried will be referred to as the closed conformation. More specifically, the side chains of Leu8, Ile44, His68 and Val70 of one moiety fit snugly onto a surface formed by the same amino acids on the other moiety (Fig. 1 ▶ *b*). Moreover, the same seven hydrogen bonds were found in each of the three distal–proximal pairs, notably between the carbonyl O atoms of Gly47 and Leu71 and the backbone amides of Leu71 and Gln49, respectively. The overall arrangement of the distal and proximal moieties is thus remarkably similar among the three Ub_2_ molecules in the asymmetric unit (Fig. 1 ▶ *c*), with C^α^ root-mean-square deviation (r.m.s.d.) values that are between 0.39 and 0.53 Å.

A previously reported crystal structure of Ub_2_ (Cook *et al.*, 1992 ▶) has a single molecule in the asymmetric unit, which also adopts the closed conformation (Fig. 1 ▶ *c*). C^α^ r.m.s.d. values of 0.68–0.89 Å were calculated between the previous structure (PDB code 1aar; Cook *et al.*, 1992 ▶) and each of the Ub_2_ subunits in the new crystal structure. The previous crystal form was obtained by crystallizing Ub_2_ in the presence of 2-methyl-2,4-pentanediol (MPD) and sodium citrate at pH 5.0, instead of PEG 4000, Li_2_SO_4_ and Tris at pH 8.5 as used in the current study. Despite these different conditions, the same set of hydrophobic interactions and hydrogen bonds were found as in the previous Ub_2_ crystal structure. The closed conformation was also observed in one of the crystal forms of Ub_4_ (Phillips *et al.*, 2001 ▶) but not in the other (Cook *et al.*, 1994 ▶). Similar to the case reported here, the more recent Ub_4_ crystal structure was obtained from a crystal grown in the presence of a peptide derived from a ubiquitin-binding protein (S5a), which was not incorporated into the crystal but yielded Ub_4_ crystals in a different space group (Phillips *et al.*, 2001 ▶). NMR residual dipolar couplings and relaxation-anisotropy studies have shown that the closed conformation of Ub_2_ predominates in solution at pH values above 6.8 and is in rapid equilibrium with an open form (Varadan *et al.*, 2002 ▶). The solution structure of the closed conformation, which was determined by a docking approach using chemical shift perturbation data and residual dipolar coupling restraints (PDB code 2bgf; van Dijk *et al.*, 2005 ▶), superposes with an average C^α^ r.m.s.d. of ∼1.5 Å with the three Ub_2_ conjugates observed in the present crystal structure. This shows that the overall arrangement of the Ub_2_ conjugate in the crystal is similar to that observed in solution.

**Figure 2:**
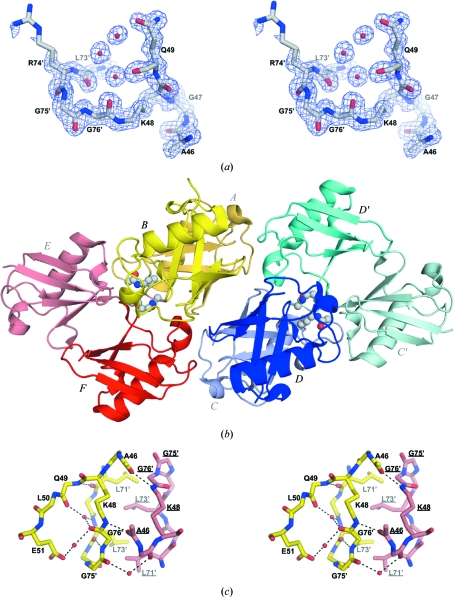
Conformation of the isopeptide bond in the crystal structure of Ub_2_. (*a*) Cross-eye stereoview of the σ_A_-weighted 2*F*_o_ − *F*_c_ electron-density map at the isopeptide linkage contoured in blue at 0.35 e Å^−3^. The atomic model is drawn as sticks. Water molecules are drawn as red spheres. (*b*) The three Lys48-linked Ub_2_ molecules in one asymmetric unit are coloured yellow for chains *A*–*B*, blue for chains *C*–*D* and red for chains *E*–*F*. Distal (*A*, *C* and *E*) and proximal (*B*, *D* and *F*) ubiquitin moieties are distinguished by pale and dark shades, respectively. Chains *C*′ and *D*′ are from an adjacent asymmetric unit and are labelled in pale and dark cyan, respectively. The isopeptide linkages are shown as spheres coloured by atom type (white, carbon; blue, nitrogen; red, oxygen). (*c*) Cross-eye stereoview of the isopeptide bond and its interactions. Residues labelled with primes belong to a distal moiety. Hydrogen bonds are shown as dashed lines. C atoms of chains *A*–*B* and *E*–*F* are shown in yellow and salmon red, respectively.

**Figure 3:**
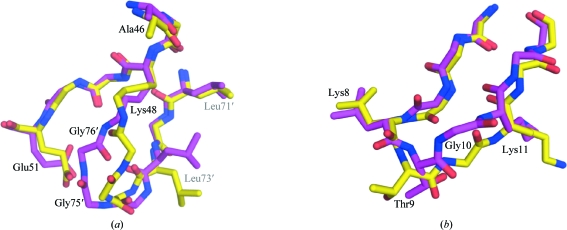
Comparison of loop conformations in different Ub_2_ crystal structures. (*a*) Comparison of the isopeptide-bond conformation in the two Ub_2_ crystal structures. Chains *A*–*B* of the new crystal structure are coloured yellow and the previous structure (PDB code 1aar; Cook *et al.*, 1992 ▶) is coloured magenta. Residues labelled with primes belong to a distal moiety. The conformation of the isopeptide bond in chains *C*–*D* and *E*–*F* is similar to that in chains *A*–*B*. (*b*) Comparison of the β1–β2 loop conformation in chain *B* (yellow) and the previous crystal structure (magenta). The conformation of this loop in chains *C*–*D* and *E*–*F* of the new structure is similar to that shown in magenta.

Although Ub_2_ adopts the closed conformation in both crystal forms (this study and Cook *et al.*, 1992 ▶), differences are observed in the configuration of the isopeptide linkage. Well defined electron density was observed for the isopeptide linkage in the new crystal structure (Fig. 2 ▶ *a*), with *B* factors near main-chain levels for the atoms involved (between 15 and 25 Å^2^, compared with 10–20 Å^2^ for main-chain atoms). This contrasts with the previously published Ub_2_ crystal structure, which showed slight disorder for these residues (*B* factors of >30 Å^2^, compared with 10–20 Å^2^ for main-chain atoms), although electron density was also visible for the isopeptide bond (Cook *et al.*, 1992 ▶). The crystal packing probably induces this order in the new crystal form, since isopeptide linkages from molecules within or between different asymmetric units make a number of reciprocal interactions (Fig. 2 ▶ *b*). The ∊-amide group of Lys48 in the distal subunit (involved in the isopeptide bond) makes a hydrogen bond to the backbone carbonyl O atom of Ala46 in a neighbouring subunit and the side chain of Leu73 in the proximal subunit intercalates between Leu71 and Leu73 in the neighbouring subunit (Fig. 2 ▶ *c*). These interactions were not observed in the previous structure owing to different crystal packing. A network of intramolecular hydrogen bonds and water molecules that were not observed in the previous crystal structure further stabilizes the isopeptide-linkage conformation. A water molecule makes hydrogen bonds to the carbonyl O atoms of Gly76 and Gln49 in the distal and proximal moieties, respectively, and another water molecule bridges the side chain of Glu51 with the carbonyl O atom of Gly76 (Fig. 2 ▶ *c*). Finally, the carbonyl O atom of Leu73 makes a hydrogen bond to the amide group of Gly76 in the distal moiety. These interactions were observed in all three isopeptide linkages in the asymmetric unit, which thus adopt nearly identical conformations with residues 73–76 (distal) and Lys48 (proximal) forming a long U-shaped loop (Fig. 1 ▶ *c*). The con­formation of the isopeptide linkage in the previous structure is similar, but shows significant differences in the backbone torsion angles for residues 73–76 (Fig. 3 ▶ *a*). The isopeptide bond is in a *trans* configuration in both crystal structures, but the carbonyl O atom of Gly76 points in opposite directions, which imposes a reconfiguration of Gly75 and Gly76. This emphasizes the flexibility of the isopeptide linkage, which is essential for the function of Ub_2_ because ubiquitin-binding domains need to access the hydrophobic patches of ubiquitin that are occluded in the closed conformation (Fig. 1 ▶ *a*). Solution NMR dynamics studies have indeed shown that the closed conformation of Ub_2_ experiences fast interdomain motion on a 10 ns timescale (Ryabov & Fushman, 2006 ▶).

Additional differences are found in the backbones of different Ub_2_ subunits, notably at the free C-termini of the proximal moieties (*B*, *D* and *F*), which show variable levels of disorder for residues Arg74–Gly76 (Fig. 1 ▶ *c* and Table 1 ▶). The loop residues Thr9 and Gly10, which are located between the β1 and β2 strands, also adopt a different con­formation in chain *B* compared with the other chains (Figs. 1 ▶ *c* and 3 ▶ *b*) and the electron density around these residues is weaker in chain *B* in comparison with the other chains. In the previous crystal structure this loop adopts the conformation observed in chains *A*, *C*, *D*, *E* and *F* in the new crystal structure. Interestingly, the chemical environment around Thr9 and Gly10 is nearly identical for all chains, including chain *B*, with Thr9 being in proximity to Ala46/Gly47 and Ser57/Asp58 in two different neighbouring subunits (not shown). This suggests that the two conformations observed have similar potential energy, with the most frequent being slightly more stable. This loop shows significant backbone dynamics in solution (Lakomek *et al.*, 2006 ▶), which is consistent with the variability observed here.

## 4. Conclusions

A new crystal form of Lys48-linked Ub_2_ was obtained and its structure was determined by X-ray crystallography to 1.6 Å resolution. The asymmetric unit is composed of three Ub_2_ molecules that all adopt the closed conformation, as observed in solution (Varadan *et al.*, 2002 ▶) and in the previous crystal structure (Cook *et al.*, 1992 ▶), despite the different crystallization conditions and crystal packing. The new crystal form reveals a new conformation for the isopeptide linkage, which interacts with other isopeptide linkages in the other subunits. A new conformation was also observed for the loop between the β1 and β2 strands. These local differences emphasize the flexibility of the isopeptide linkage and the β1–β2 loop.

## Supplementary Material

PDB reference: Lys48-linked diubiquitin, 3m3j

## Figures and Tables

**Table 1:** X-ray data-collection and refinement statistics for Ub_2_ Values in parentheses are for the last shell.

X-ray source	ESRF ID29
Wavelength (Å)	0.97625
Space group	*C*2
Unit-cell parameters (Å, °)	*a* = 58.7, *b* = 78.7, *c* = 93.1, α = γ = 90, β = 97.9
Mosaicity (°)	0.30
Images	180
Oscillation angle (°)	1.0
Resolution (Å)	39.90–1.60 (1.69–1.60)
Unique reflections	54118 (7792)
Completeness (%)	97.9 (96.8)
Multiplicity	3.8 (3.8)
〈*I*〉/〈σ(*I*)〉	16.1 (3.2)
*R*_merge_†	0.057 (0.432)
Solvent content (%)	41
No. of reflections in *R*_free_ set (5%)	2738
*R*_work_	0.183
*R*_free_	0.229
FOM	0.851
R.m.s. deviations from ideal values‡	
Bond lengths (Å)	0.012
Bond angles (°)	1.5
Torsion angles (°)	6.1
Protein atoms	3962
Water atoms	360
Ligand atoms (1 ethylene glycol, 3 sulfate ions)	19
Disordered residues (not modelled)	Chain *B*, 76; chains *D*, *F*, 74, 75, 76§
Average *B* factors (Å^2^)	
Protein main chain	19
Protein side chain	21
Water	32
Ethylene glycol	28
Sulfate ions	58
Ramachandran outliers¶	1 [Gln62 in chain *D*]
Estimated coordinate error†† (Å)	0.18
PDB code	3m3j

†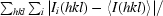 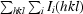, where *I_i_*(*hkl*) is the intensity of the *i*th measurement of reflection *hkl* and 〈*I*(*hkl*)〉 is the mean value for all *i* measure­ments.

‡ Ideal values as reported in Engh & Huber (2001 ▶).

§ These residues correspond to the C-termini of proximal ubiquitin moieties.

¶ Residues for which the backbone torsion angles are outside the core region of the Ramachandran plot (Kleywegt & Jones, 1996 ▶).

†† Coordinate error estimated from a Luzzati plot (*R*/*R*_free_ *versus* resolution) as reported by *SFCHECK* (Vaguine *et al.*, 1999 ▶).
